# High-density lipoprotein mimetic nano-therapeutics targeting monocytes and macrophages for improved cardiovascular care: a comprehensive review

**DOI:** 10.1186/s12951-024-02529-x

**Published:** 2024-05-17

**Authors:** Juan Zhen, Xiangjun Li, Haitao Yu, Bing Du

**Affiliations:** 1 The First Hospital of Jilin University, Changchun, 130021 China; 2 School of Pharmaceutical Science, Jilin University, Changchun, 130021 China

**Keywords:** Cardiovascular diseases; nanotechnology, Nanomedicine, High-density lipoprotein, Targeted therapy, Monocytes, Macrophages

## Abstract

The prevalence of cardiovascular diseases continues to be a challenge for global health, necessitating innovative solutions. The potential of high-density lipoprotein (HDL) mimetic nanotherapeutics in the context of cardiovascular disease and the intricate mechanisms underlying the interactions between monocyte-derived cells and HDL mimetic showing their impact on inflammation, cellular lipid metabolism, and the progression of atherosclerotic plaque. Preclinical studies have demonstrated that HDL mimetic nanotherapeutics can regulate monocyte recruitment and macrophage polarization towards an anti-inflammatory phenotype, suggesting their potential to impede the progression of atherosclerosis. The challenges and opportunities associated with the clinical application of HDL mimetic nanotherapeutics, emphasize the need for additional research to gain a better understanding of the precise molecular pathways and long-term effects of these nanotherapeutics on monocytes and macrophages to maximize their therapeutic efficacy. Furthermore, the use of nanotechnology in the treatment of cardiovascular diseases highlights the potential of nanoparticles for targeted treatments. Moreover, the concept of theranostics combines therapy and diagnosis to create a selective platform for the conversion of traditional therapeutic medications into specialized and customized treatments. The multifaceted contributions of HDL to cardiovascular and metabolic health via highlight its potential to improve plaque stability and avert atherosclerosis-related problems. There is a need for further research to maximize the therapeutic efficacy of HDL mimetic nanotherapeutics and to develop targeted treatment approaches to prevent atherosclerosis. This review provides a comprehensive overview of the potential of nanotherapeutics in the treatment of cardiovascular diseases, emphasizing the need for innovative solutions to address the challenges posed by cardiovascular diseases.

## Introduction

Globally, cardiovascular diseases are the primary reason for mortality, taking the lives of approximately 17.9 million people each year, with an overall 31% contribution to all fatalities. Coronary heart disease (CHD), in particular, is still one of the main reasons for deaths, disease, and disabilities around the world. Over the past several decades, the deployment of therapeutic and preventive strategies targeted at managing cardiovascular diseases (CVDs) has resulted in a constant drop in mortalities and increased life expectancies [1, 2]. Several treatment regimens have been developed to address high CVD risk factors and decrease their negative effects. Notably, recent interest has been focused on the concept of a ‘polypill’—a prescription comprising low amounts of numerous medications. The broad prevalence of CVD risk factors and the efficiency of pharmacologic therapies, such a combination might result in an 88% reduction in CVD deaths. CVD includes condition such as coronary artery disease, stroke, heart failure, and others. CVD kills nearly 4 million people in Europe each year, accounting for 45% of all documented deaths. According to the literature, the number of Disability Adjusted Life Years (DALYs) lost for CHD was 11.8 million and 24.2 million for cerebrovascular diseases [3]. In recent times, the prevalence of chronic diseases has become increasingly prominent as significant contributors to overall global mortality [4].

**Fig. 1:**
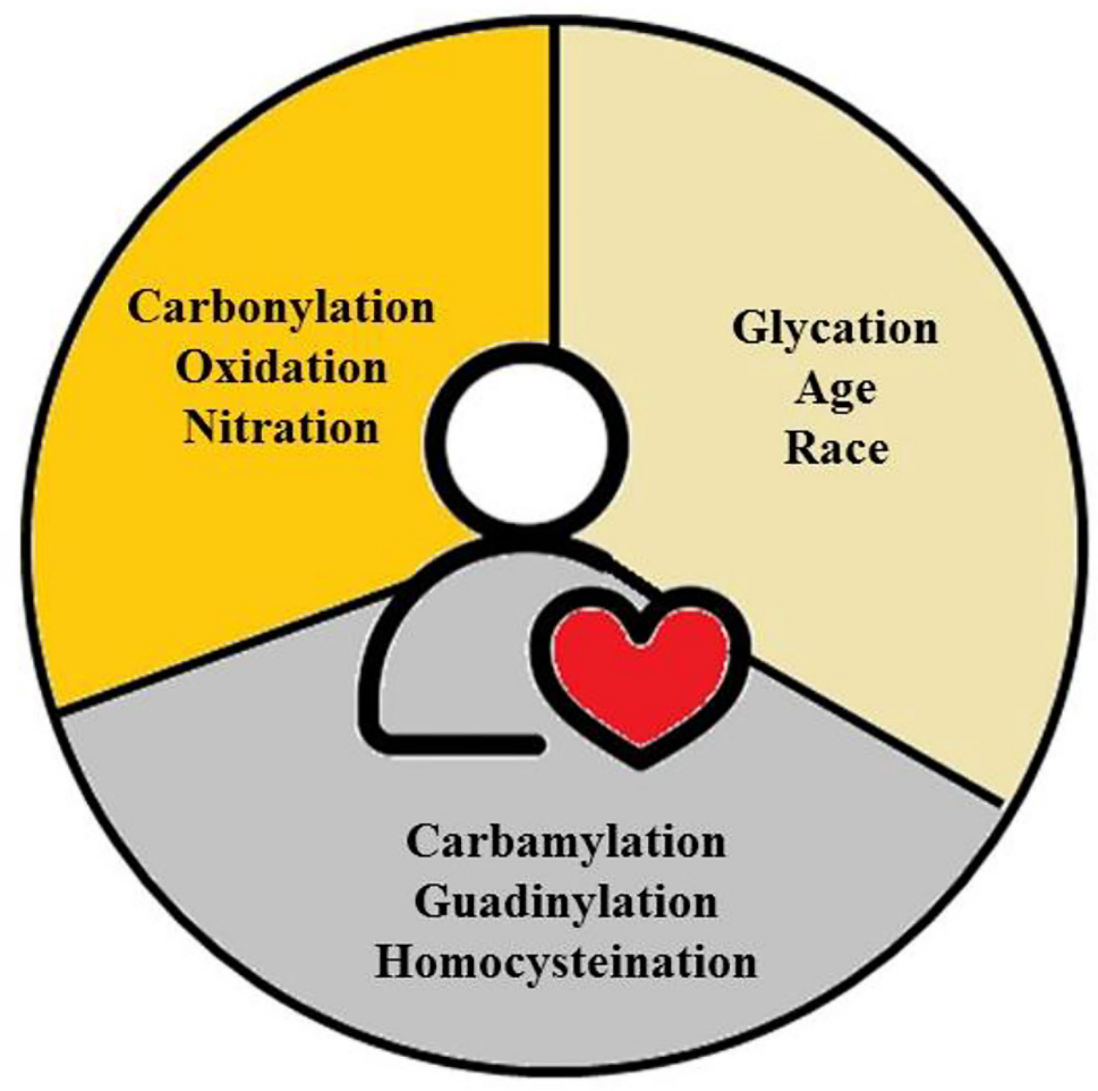
Illustration of the multifaceted reasons behind the increasing prevalence of cardiovascular diseases, emphasizing the complex interplay of lifestyle, environment, genetics, socioeconomic status, and emerging health issues

The prevalence of angina, acute myocardial infarction, and sudden death—the three basic clinical manifestations of CHD—has been studied epidemiologically, with variations based on individual-level risk factors, age, gender, and ethnicity. These differences go beyond individual cases to include patterns among countries, regions, and social strata within nations at the population level. Furthermore, the occurrence rates of these cardiovascular events vary significantly over time [5]. Researchers predict that by 2030, non-communicable diseases will account for more than 75% of global mortality. In low-income countries, CVDs are predicted to outnumber infectious diseases such as HIV/AIDS, TB, and malaria, as well as maternal and perinatal problems and nutritional disorders combined. According to the WHO, CVD is the leading cause of death globally. It accounted for 31% of all global deaths in 2016. The Global Burden of Disease Study (GBD) provides comprehensive data on the prevalence and impact of CVD globally. CVD exerts a significant economic burden on individuals and healthcare systems, including direct medical expenditures as well as indirect costs associated with lost productivity [6]. CVD and its risk factors can negatively affect one’s quality of life, influencing everyday activities and general well-being [7], as illustrated in Fig. 1.

**Fig. 2:**
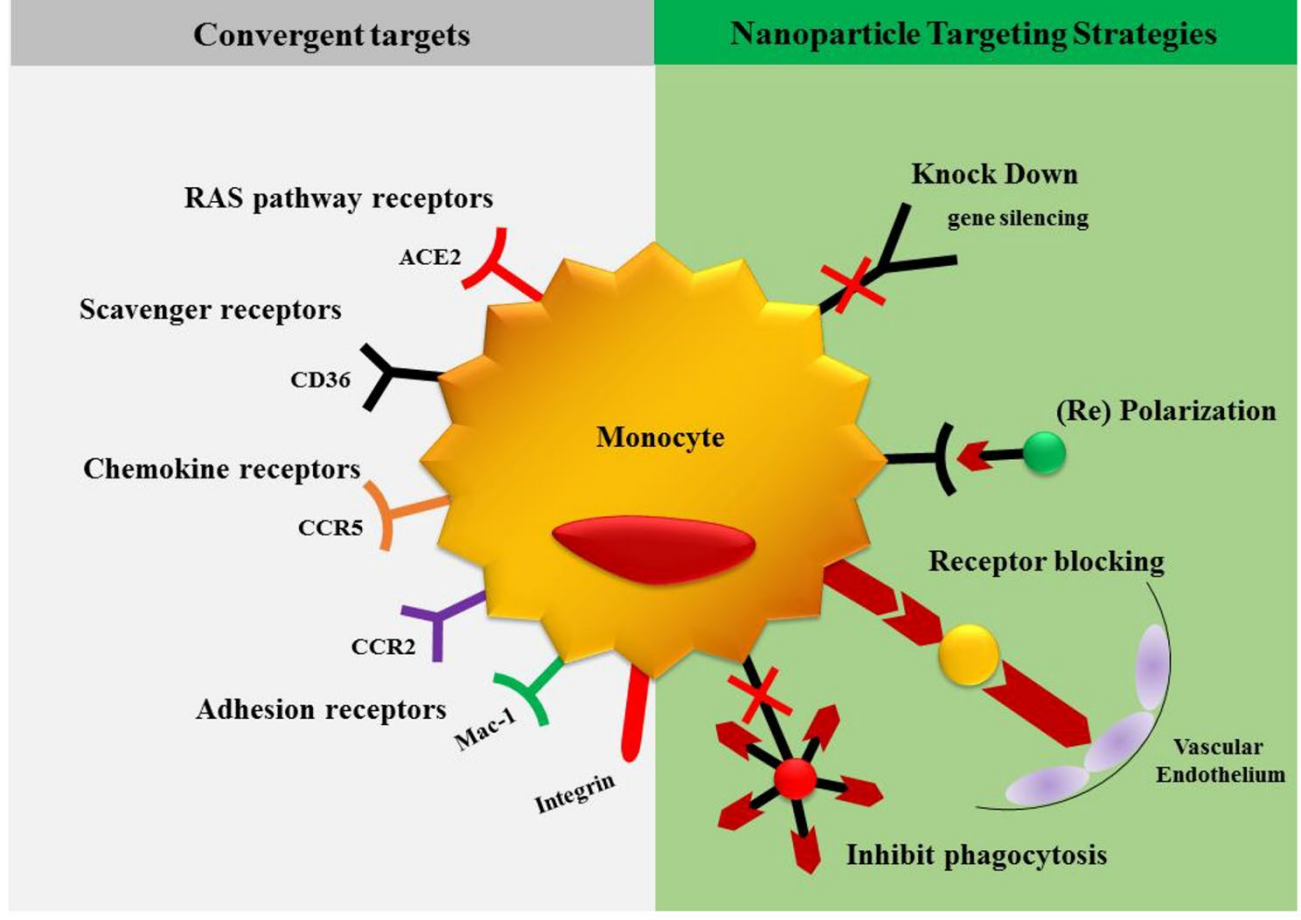
Monocytes serve as a focal point for nanoparticle therapeutic intervention in cardiovascular diseases

Monocytes and macrophages are essential components of the vertebrate innate immune system, having diverse tasks in both homeostasis and disease situations, as shown in Fig. 2. Innate immunity has a key role in both the first response and the prolonged phase of heart damage, according to most experts. In the human setting, the expression levels of CD14 and CD16 define three monocyte subsets: classical (CD14^++^ CD16), intermediate (CD14^++^ CD16^+^), and non-classical (CD14^+^ CD16^++^) [8]. Ly-6 C expression differentiates mature murine monocytes into two groups. Ly-6C^high^ (CCR2)^high^ (CX_3_CR1l)^low^ monocytes have a penchant for aggregation at inflammatory regions like acute myocardial infarction (MI), where they develop into macrophages. Non-classical Ly-6C^low^ CCR2^low^ CX3CR1 high monocytes, on the other hand, patrol the endothelium to maintain homeostasis [9]. On the other hand, macrophages are the most prevalent immune cells in the heart in normal physiological settings. These cells have a spindle-like shape and are found in the interstitial space or near endothelial cells [10, 11]. Until recently, the prevailing belief held that macrophages, over the last fifty years, originated exclusively from circulating blood monocytes. Recent investigations employing genetic fate mapping, parabiosis, and adoptive transfer methodologies have revealed a different narrative. These studies demonstrate that macrophages residing in tissues such as the brain, liver, lung, and skin are not sourced from circulating monocytes. Instead, they undergo replenishment through local proliferation [12–14].

Macrophages have an important role in the physiology of numerous cardiovascular disorders, including atherosclerosis and aortic aneurysms. Long-term local inflammatory reactions are brought on by their buildup in the arterial wall and involve the release of chemokines, cytokines, and enzymes that break down matrix proteins [15]. Atherosclerotic cardiovascular disease and aortic aneurysm are two distinct diseases. Whereas medial damage distinguishes an aortic aneurysm from atherosclerosis, lipid-rich plaques are present in the subintimal area [16, 17]. Clinically, these two main vascular diseases have significant risk factors, including a masculine propensity, smoking, high blood pressure, and hypercholesterolemia [18, 19]. A significant pathogenic characteristic shared by atherosclerosis and aortic aneurysm is macrophage accumulation in the arterial wall [20, 21]. Furthermore, macrophages interact dynamically with vascular cells and are responsible for phenotypic alterations in vascular smooth muscle cells [22, 23]. Macrophages are a major source of enzymes that break down matrix proteins; they are crucial for the rupture of atherosclerotic plaques and the deterioration of elastin integrity in aneurysmal aortas [24].

The quantity of circulating monocytes is directly correlated with cholesterol levels [25]. During inflammation, monocyte numbers rise and are crucial for host defense [26]. The degree of the infarct and the severity of the disease are correlated with the number of monocytes in cardiovascular diseases [27–29]. In humans, CD14 + + CD16 monocytes have a negative correlation with post-MI left ventricular function, but intermediate CD14 + + CD16 + monocytes have a positive correlation with cardiovascular disease severity [28, 30, 31]. Monocytes, particularly classical monocytes, have a significant part in the production of atherosclerotic plaques, a characteristic of CVDs. In response to inflammatory stimuli such as oxidized LDL, these cells migrate into the subendothelial region of arteries. When monocytes enter the plaque, they convert into macrophages, which absorb oxidized LDL particles and turn into foam cells, which contribute to plaque expansion [25]. While this inflammatory mechanism was designed to protect against pathogens, it can be harmful in the context of chronic cardiovascular disease. Monocytes contribute to cardiovascular health through their roles in tissue regeneration and angiogenesis, in addition to their involvement in atherosclerosis. Monocytes can develop into reparative macrophages, which aid in the resolution of inflammation and promote tissue healing [11]. Furthermore, monocytes participate in angiogenesis, or the development of new blood vessels, which is necessary for restoring blood flow to damaged tissues such as the ischemic myocardium.

Triglyceride, phospholipid, cholesterol transport and metabolism are facilitated by HDL, which are endogenous, nano-sized protein-lipid particles that circulate in the blood [32, 33]. Among the well-established functional features of HDL, athero-protective properties stand out. These include its well-known antioxidant and anti-inflammatory properties, as well as its role in endothelial cell function maintenance. It is also noteworthy because HDL plays a crucial role in mediating cholesterol efflux, which is essential for promoting cellular cholesterol outflow and reverse cholesterol transport (RCT). This is usually regarded as HDL’s most important anti-atherogenic property. This has led to a lot of concern about the possible cardiovascular advantages of reconstituted apolipoprotein A-I (ApoA-I), the primary protein ingredient of HDL. The goal of these studies is to see if such therapies can reduce coronary events in people with CVD [32, 34, 35].

HDL mimetic nanotherapeutics with precision targeting capabilities can selectively interact with monocytes, harnessing natural HDL’s anti-inflammatory qualities. These nanotherapeutics, which mirror the effects of HDL, can modify monocyte behavior, decreasing inflammatory responses and facilitating atherosclerotic lesion regression. The development of nanotherapeutics with specific monocyte targeting presents an intriguing route for therapies aimed at reducing atherosclerosis-associated cardiovascular problems [36]. HDL mimic nanotherapeutics can be designed to engage with macrophages selectively, increasing their anti-inflammatory M2 phenotype and enhancing processes like cholesterol efflux. This targeted modification of macrophage function is consistent with HDL’s athero-protective capabilities and may help to stabilize atherosclerotic plaques. The development of nanotherapeutics customized particularly for macrophage targeting holds great promise for developing precision medicine methods in cardiovascular health [37].

This comprehensive review addresses the imperative need for advancements in cardiovascular care by focusing on HDL mimetic nano-therapeutics targeting monocytes and macrophages. Given the escalating prevalence of CVDs, there is a critical demand for innovative interventions to overcome existing limitations. HDL mimetic nano-therapeutics offer a unique approach with precise targeting of monocytes and macrophages, pivotal components in CVD pathophysiology. By consolidating current research, this review aims to provide a succinct yet insightful overview, shedding light on mechanisms, challenges, and future potentials. The synthesis intends to serve as a valuable resource for researchers, clinicians, and policymakers, fostering further advancements in this promising field.

### Biology of monocytes and macrophages

The mononuclear phagocyte system associated with innate immunity includes monocytes and macrophages [38]. Monocytes are leukocytes that circulate in the blood and spleen and are derived from bone marrow. Their capacity to identify “danger signals” using pattern recognition receptors makes them unique. In response to infection and damage, monocytes can release chemokines, present and phagocytose antigens, and multiply [39]. Monocytes can differentiate into dendritic and macrophage cells once they are recruited to tissues. However, macrophages are typically thought of as terminally differentiated cells that migrate to local lymph node beds via lymphatics and processed antigens [40]. In order to draw in additional immune cells, they also release chemokines and phagocytose infections or toxins [41].

### Monocytes and macrophages in the cancer microenvironment

Although monocytes and macrophages play a unique part in immunity and tissue homeostasis, they also contribute to a wide range of pathologies, making them desirable targets for therapeutic intervention. A thorough understanding of these cells’ origins and the mechanisms preserving their homeostasis will be necessary for any potential intervention strategies that attempt to manipulate them. According to recent findings, monocytes do not contribute significantly to the tissue macrophage populations during some types of inflammation or in a steady state [42]. Instead, the majority of mouse tissue macrophage populations are seeded before birth, originate from embryonic precursors, and can sustain themselves as adults through self-renewal. Cell differentiation, a fundamental process in biology, explores how cells originating from a single fertilized egg specialize into unique structures and functions [43]. Stem cells, with the capacity for infinite division and specialization under specific conditions, play a pivotal role in this process. While all somatic cell genomes are identical, variations in gene expression occur as different cell types express unique combinations of genes at different times. This dynamic gene expression, regulated by transcription factor proteins that either promote or inhibit gene transcription, determines the diverse morphological and physiological properties of cells [44]. The ability of cells in the human body to specialize into numerous distinct cell types is attributed to the coordinated activity of these transcription factors. Understanding cell differentiation is crucial for unraveling the complexities of development and tissue function [45].

A collection of cells with a similar shape and function is referred to as a tissue in simple terms. They constitute an intermediary cellular organizational level situated between the organ system and the cells. Tissue functional groups are then combined to form organs [46]. The body’s tissues perform distinct functions according to their specific types. For instance, nerve and muscle tissues carry signals, respectively, and epithelial tissues cover and shield surfaces [47]. There can be significant functional differences in biological processes between tissues due to tissue-specific gene expression, which can influence the presence or absence of specific protein complexes and interactions [48].

### Monocytes and macrophages in the cardiovascular system

Macrophages, are essential for both immunity and the control of organ growth and functioning [49]. These cells, found in all tissues, remove infections, remove dead cells, give T cells antigens, and release cytokines to indicate ongoing tissue damage and aid in tissue repair. Significant populations of tissue-resident macrophages have been found in healthy tissues using recent techniques; these cells are especially prevalent in the heart and blood vessels [50]. Both the quantity and phenotype of these cells significantly alter under pathological circumstances. Although the majority of steady-state macrophages do not require monocytes for survival, those present in inflammatory vascular walls and diseased hearts are derived from hematopoietic organs [50]. Recognizing the signals that regulate macrophage supply and function, employing imaging techniques to track changes in macrophage phenotype and quantity, and investigating ways to modify macrophage biology offer potential avenues for controlling cardiovascular inflammation [51].

Numerous nutrients, either pro- or anti-inflammatory, regulate inflammation. The use of diets that are either rich in or deficient in a number of specific nutrients to reduce inflammation has thus been the subject of extensive research [52]. This suggests that the dietary balance of nutrients should be taken into account and that there should be another lever to manage the inflammatory process. Oxidative stress and inflammation are closely related. If either occurs excessively or for an extended period, it can result in systemic inflammatory response syndrome (SIRS), which includes septic shock and multi-organ failure [53]. Additionally, they have a close relationship with insulin sensitivity and wound healing. This article concentrates on the research on inflammation or, in certain situations, the result (morbi-mortality) when the cause is probably inflammation [54, 55].

### Uncovering the role of monocytes and macrophages in atherosclerosis

Atherosclerosis, a leading cause of global mortality and disability, is characterized by lipid accumulation, inflammation, local neo-angiogenesis, and apoptosis within arterial walls [56]. Monocytes and macrophages, integral components of the innate immune system, play a pivotal and novel pathogenic role in the initiation and progression of atherosclerosis, particularly in relation to neovascularization [57]. While existing data predominantly originates from animal studies, an increasing body of evidence confirms the specific involvement of these mononuclear cell subtypes in human atherosclerosis [58]. This review aim for the current knowledge on the roles of monocytes and macrophages in human atherosclerosis, emphasizing their potential as biomarkers and their correlation with plaque characteristics such as neo-angiogenesis, offering insights into future targeted treatments to mitigate cardiovascular disease risks.

#### Mechanisms of monocyte recruitment

Originating in bone marrow, monocytes travel through the bloodstream to peripheral tissues, where local factors, pro-inflammatory cytokines, and microbic products influence their differentiation into macrophages or dendritic cells. Recruited monocytes are important for fighting infections, but they also play a role in degenerative and inflammatory disease [59]. The complex processes governing monocyte trafficking during inflammation, infections, and homeostasis are still being studied. Interestingly, neointimal growth is linked to the inflammatory response to acute vessel wall damage; feverish monocyte infiltration exacerbates this process and may even encourage restenosis. The adhesion of monocytes to the site of mechanical injury is a crucial step in the recruitment process [60].

#### Macrophage polarization dynamics in atherosclerotic lesions

The polarity and metabolic characteristics, macrophages are important in the initial, proliferative, rupture, and healing stages of atheromatic plaque development in atherosclerosis. Key factors influencing the growth of atheromatic plaque are modifications to macrophage phenotype and metabolism related to hypoxia, which are mediated by HIF-1α expression [61]. Atherosclerosis, a chronic inflammatory condition that builds up fat deposits in blood vessel walls, raises the risk of heart attacks and strokes [62]. Numerous variables, including as age, genetics, lifestyle, and underlying medical disorders, might influence atherosclerosis. While unstable atherosclerotic plaques raise the risk of thrombotic and embolic complications, stable plaques grow slowly and have a low embolic potential. Their high degrees of variability and plasticity within plaques, macrophages contribute to the complex stages of atherosclerosis, influencing the microenvironment in ways such as hyperlipidemia, cytokine hyper-activation, hypoxia, apoptosis, and necroptosis [63, 64].

### High-density lipoprotein (HDL) and cardiovascular diseases

HDL is sometimes called “good” cholesterol since it is essential in removing excess cholesterol from the circulation and processing it to the liver for elimination [65]. Considered by many to be the master of lipid metabolism, HDL is essential for coordinating a multitude of processes that support cardiovascular health [66]. Reverse cholesterol transport, which includes taking excess cholesterol out of peripheral tissues and transferring it to the liver for excretion, is its primary function [67]. Beyond controlling cholesterol, HDL plays a virtuoso role in preventing atherosclerosis and CVDs by exhibiting its anti-inflammatory and antioxidant qualities [68]. Nonetheless, recent studies indicate that HDL’s efficacy might not be determined solely by quantity; HDL particles’ functionality and quality are just as important. Understanding changes as the scientific melody progresses. However, recent research suggests that the quantity alone may not dictate HDL’s effectiveness; the quality and functionality of HDL particles are equally critical [69].

**Fig. 3:**
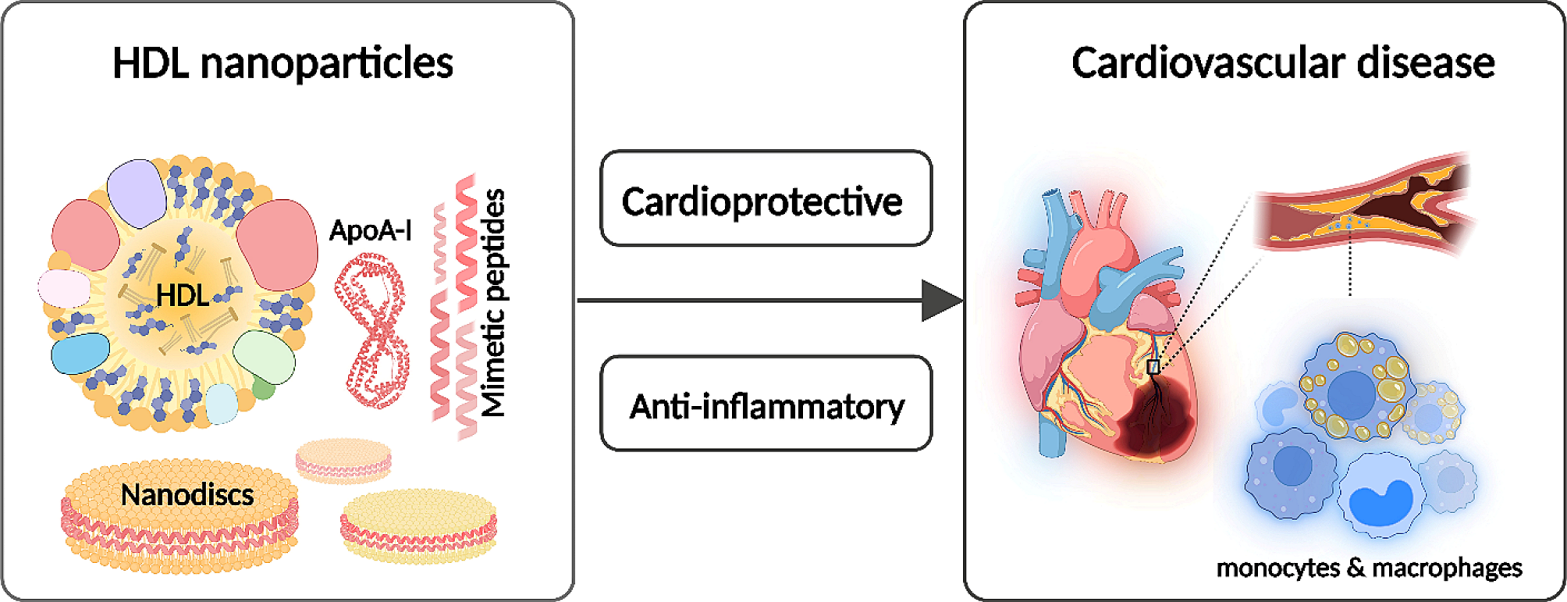
The distinctive features of HDL nanoparticles and their promising applications in cardiovascular diseases, including their unique structure, capacity for drug delivery, anti-atherosclerotic effects, imaging capabilities, and potential future directions in research and development. Adapted from [72] under the terms and conditions of the Creative Commons Attribution (CC BY) license (https://creativecommons.org/licenses/by/4.0/)

Furthermore, HDL has many functions, including powerful anti-inflammatory effects in addition to lipid metabolism. Through lowering oxidative stress, inhibiting the expression of adhesion molecules, and adjusting immune responses, HDL plays a critical modulatory role in reducing inflammation. As of its anti-inflammatory properties, HDL plays a crucial role in preserving vascular health, which has implications for cardiovascular disease. Investigating the molecular details of HDL’s anti-inflammatory properties contributes to our understanding of the variety of roles that HDL plays in health and highlights the potential for novel approaches to the treatment of cardiovascular conditions associated with inflammation. It also opens up promising avenues for targeted therapies [70, 71], as illustrated in Fig. 3.

### The multifaceted functions of HDL in cardiovascular health

HDL, in addition to its traditional role in cholesterol metabolism, demonstrates a variety of complex functions that intricately contribute to cardiovascular health. The range of benefits that HDL offers includes antioxidant and anti-inflammatory properties, stabilization of plaque, enhancement of endothelial function, and even possible roles in immune response modulation. The diverse range of functions, HDL is seen as an essential component in the prevention of atherosclerosis and cardiovascular disorders. Although HDL has historically been seen through the prism of cholesterol control, its importance in preserving cardiovascular health is highlighted by an awareness of its broader effects on vascular health. Deciphering these complex roles contributes to our understanding of the complex biology of HDL and provides new directions for potential treatments of cardiovascular diseases [73].

One of the most important aspects of vascular health is maintaining and repairing the endothelium, or inner lining of blood vessels, and high-density lipoprotein, or HDL, becomes an important component of this process. HDL has protective effects on the endothelium in addition to its widely recognized function in the transport of cholesterol. By increasing the synthesis of nitric oxide, which is essential for vasodilation and preserving vascular homeostasis, it supports endothelial function. Furthermore, HDL has anti-inflammatory qualities that lower oxidative stress and endothelial inflammation. Together, these activities support the maintenance and repair of the endothelium, which is essential in halting the development of cardiovascular disease and vascular dysfunction. Comprehending the complex mechanisms through which HDL promotes endothelial health provides a valuable understanding of potential therapeutic approaches for conditions exhibiting endothelial dysfunction and damage [74, 75].

### Role of HDL in atherosclerosis and cardiovascular disease

HDL plays a vital role in preventing atherosclerosis and goes above and beyond its traditional function in controlling cholesterol to maintain arterial integrity. HDL demonstrates a variety of functions that work together to maintain the health of the arteries. As a strong antioxidant, HDL prevents oxidative damage, stops LDL cholesterol from oxidizing, and reduces lipid peroxidation. Furthermore, HDL inhibits the formation of blood clots and modifies platelet function to exhibit anti-thrombotic properties. These defense mechanisms highlight the role HDL plays in preventing the development and advancement of atherosclerosis, establishing it as an essential element in preventing arterial integrity and averting cardiovascular diseases. Comprehending and utilizing the multifaceted functions of HDL present encouraging opportunities for therapeutic approaches targeted at maintaining arterial integrity and reducing the consequences of atherosclerosis [73]. An important step in the development of atherosclerosis is the oxidation of LDL, which is effectively inhibited by HDL. One important factor in the development and course of atherosclerotic plaques is oxidized LDL. As a protective factor, HDL stops LDL cholesterol from oxidizing. In order to prevent the production of oxidized LDL, it interacts directly with LDL particles and eliminates lipid hydroperoxides. A crucial component of HDL’s anti-atherosclerotic actions is the inhibition of LDL oxidation it mediates, underscoring the role HDL plays in preserving vascular health and averting cardiovascular disease. Gaining knowledge of the molecular mechanisms underlying this process could help develop targeted treatment approaches to prevent atherosclerosis [73, 76].

Furthermore, HDL inhibits processes that may contribute to plaque instability by its anti-inflammatory properties. It also helps to stabilize plaque by promoting endothelial health. The significance of HDL in maintaining vascular integrity, preventing plaque destabilization, and lowering the risk of adverse cardiovascular events associated with atherosclerosis is highlighted by these diverse actions taken together. Improving the processes by which HDL increases the stability of plaque offers hope for future therapeutic interventions in heart diseases [73, 76], illustrated in Fig. 2.

Moreover, current study emphasizes other functions of HDL in cardiovascular well-being. HDL has been discovered to facilitate reverse cholesterol transport, a vital procedure for eliminating excess cholesterol from peripheral tissues and delivering it back to the liver [77] The anti-atherogenic feature of HDL emphasizes its crucial role in avoiding the development of plaque and lowering the likelihood of cardiovascular events. Moreover, recent research indicates that HDL may have vasoprotective properties by facilitating endothelial regeneration and increasing vasodilation, hence enhancing arterial well-being and functionality [77].

Furthermore, HDL particles display diversity in their makeup and function, with subsets varying in their capacity to exert anti-atherosclerotic actions [78]. Gaining a comprehensive understanding of the unique characteristics of HDL subfractions may provide valuable knowledge about their specific functions in protecting against cardiovascular diseases and can assist in the creation of precise treatment approaches. Roles of HDL in avoiding the development of atherosclerosis and preserving the health of blood vessels highlight its potential as a target for treating cardiovascular disorders. Ongoing research into the many functions of HDL and its subfractions shows potential for the creation of innovative therapeutic approaches that target the reduction of cardiovascular disease and death rates [78].

### Nanotherapeutics in cardiovascular diseases

**Fig. 4:**
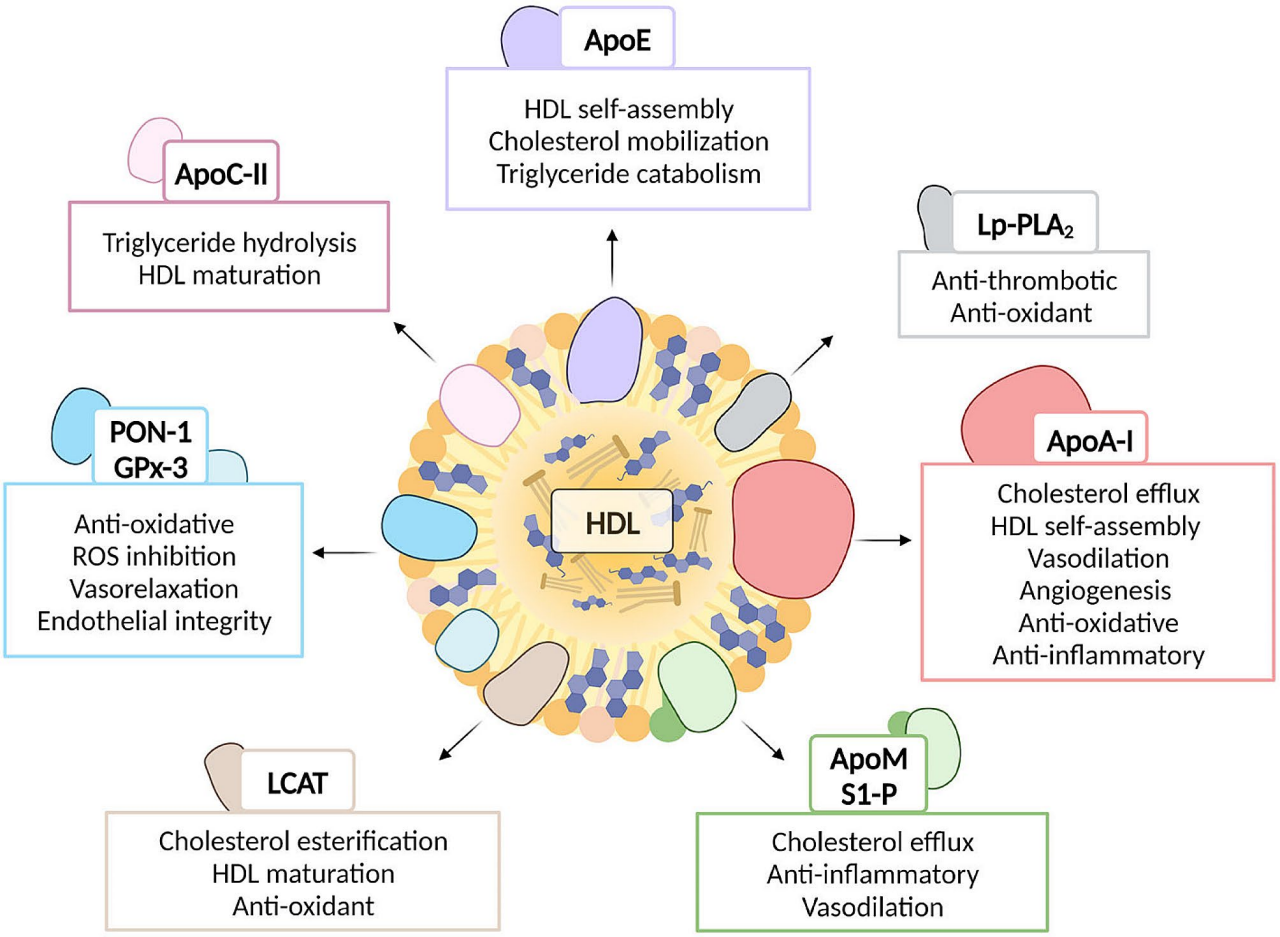
An overview of the multifaceted contributions of HDL to different parameters of human health, encompassing cardiovascular and metabolic health. Adapted from [72] under the terms and conditions of the Creative Commons Attribution (CC BY) license (https://creativecommons.org/licenses/by/4.0/)

Nanotechnology involves the manipulation of matter on an atomic and molecular scale, typically 1 to 100 nanometers. The width of the DNA helix is roughly 2 nm. To put this into context, human cells, on the other hand, have dimensions measured in microns or thousands of nanometers. Therefore, a red blood cell could include anywhere from hundreds to thousands of nanoparticles (one such nanoparticle is the hemoglobin molecule, which is roughly 5 nm in diameter). Innovative technologies are needed for the synthesis, characterization, manipulation, and imaging of these nanoparticles. Nanoparticles can be seen by techniques like atomic force microscopy and scanning tunneling microscopy, while nanostructures can be synthesized through techniques like atomic layer epitaxy and nanoimprint lithography [79]. Around the world CVDs, are thought to be a leading cause of death. As per the WHO, CVDs claimed the lives of approximately 17.9 million individuals in 2016, accounting for 31% of all deaths worldwide. Nanomedicine, refers to the use of nanotechnology to improve human health. Nanotechnology may find extensive application in the treatment of heart and vascular diseases: The focus of research can be on drug delivery (like nanoparticles that transport cardiovascular medicines), nano-devices (like nano-patterned vascular stents), or diagnosis (like implantable nano-electronic biosensors). When cardiovascular treatments are delivered by nanoparticles, for instance, the drug’s localization within the afflicted tissue may be improved, as depicted in Fig. 4. To achieve a therapeutic effect, the drug dosage and systemic exposure can be decreased by using silica, lipids, carbon nanotubes, dendrimers, proteins, and nucleic acids in nanoparticle form. These features can be engineered to enable the nanoparticles to bind to diseased tissues selectively and shield the drug from metabolism [79].

### Nanotherapeutics panorama for cardiovascular care

A malfunctioning vascular system gives rise to a wide spectrum of clinical manifestations known as CVD. Vascular dysfunction and CVD severity are causally related to reactive oxygen and nitrogen species overproduction. An appealing treatment option for diseases linked to CVD is antioxidant therapy. Overcoming obstacles related to systemic antioxidant delivery may be possible with the application of targeted nano-antioxidant therapy [80]. The most crucial aspect of managing any chronic disease or disorder is identifying the disease early on and putting the right treatment plans in place in response to the diagnostic data. About twenty years ago, a combinatorial modality that combined diagnosis and therapies gained prominence in this area. Therapy and diagnostics came together to create the combined idea of “theranostic.” The term “theranostics,” which was first used by John Funkhouser in 2002, describes a combination of suitable therapeutic interventions and diagnostic instruments grouped on a single platform. The word was coined to combine the domains of advanced applications, therapy and diagnostics [81]. In the field of cardiovascular medicine, nanotherapeutics has become a revolutionary force, providing a viable therapy pathway for a range of cardiovascular disease. Their special characteristics, nanoparticles like liposomes, dendrimers, and polymeric micelles have been used for targeted treatments, increased bioavailability, and precise drug administration. The creation of tailored medicine techniques that address the complexities of CVDs at the molecular level is made possible by these marvels of nanotechnology. There is a significant difference in the amount of research conducted on targeted monotherapies for CVD and cancer during the last five years, as evidenced by a quick search on PUBMED. On average, 11% of papers on targeted nano-therapies for cancer were also connected to CVD. On the other hand, the field of tailored nanotherapeutics for CVD treatment has been steadily expanding [80].

In 2000, the National Nanotechnology Initiative (NNI) was launched by the US government as a new project. Nanomedicine is the term for its use in the drug delivery and discovery fields. Tumor-targeted medication delivery has been the area of nano-medicine’s primary focus since its inception [82]. Advances in nanoscience and nanotechnologies have impacted almost every field of science and made life easier in the modern era. A growing field of study called nanoscience and nanotechnology deals with systems, devices, and structures that have unique characteristics and capabilities because of the way their atoms are arranged on a size of 1–100 nm. Commercial usage of nanotechnology began in the early 2000s as a result of growing public knowledge of the field and controversies surrounding it.

A more comprehensive approach to addressing the negative effects of chronic disease on health has been suggested by theranostics, which has given rise to a selective platform for the conversion of traditional therapeutic medications into specialized and customized treatments [83]. The notion of theranostics articulated its potential as customized medicines of the future in a critical manner. With high precision selectivity, it enables a sequence of sequential processes, including early disease detection and diagnosis, prognosis, therapy selection, and therapeutic efficacy monitoring [84]. Large-scale applications of spatially tailored nanomaterials have been explored in many different disciplines since the introduction of nano-technological concepts and their involvement in biological difficulties. The advent of nano-biotechnology has brought about a significant transformation in the biomedical fields and has enhanced our comprehension of various pathophysiological disorders and the treatment measures that they direct. Many characteristics, including strong surface plasmon resonance (SPR), high surface-to-volume ratio, tunability, biocompatibility, and optical and magnetic qualities, account for their importance in the diagnosis of disease and as targeted treatments [85].

### Applications of nanotherapeutics in cardiovascular diseases

CVDs are known to be one of the world’s leading causes of death. As per estimates from the WHO, 17.9 million individuals worldwide lost their lives to CVDs in 2016, making up 31% of all fatalities. Numerous cell types, including cardiac fibroblasts, cardio-myocytes, neural cells, and vascular cells, make up the heart. CVDs are caused by any abnormalities or dysfunction in these endothelial, smooth, or connective cells [86]. The general category of cardiovascular diseases, or CVDs, includes heart-related ailments such as ischemic heart disease, CVDs, arrhythmia, heart failure, stroke, vascular diseases, and cardiomyopathies. To improve disease diagnosis and therapy, the sciences of biology, biochemistry, physics, engineering, genetics, and biotechnology are combined in nanomedicine [87].

**Table 1:** This table provides a detailed overview of the various applications of nanotherapeutics in cardiovascular diseases

S.No	Application	Description	Source
1.	Monocytes and Macrophages in CVD	Monocytes contribute to cardiovascular health through their roles in tissue regeneration, angiogenesis, and atherosclerosis. They can develop into reparative macrophages, aid in the resolution of inflammation, and participate in angiogenesis, which is necessary for restoring blood flow to damaged tissues.	[88]
2.	HDL and Cardiovascular Health	HDL plays a crucial role in cardiovascular health by facilitating triglyceride, phospholipid, and cholesterol transport and metabolism. It exhibits athero-protective properties, including antioxidant and anti-inflammatory effects, and promotes endothelial cell function maintenance.	[89]
3.	Nanotherapeutics for Monocyte Interaction	HDL mimetic nanotherapeutics with precision targeting capabilities can selectively interact with monocytes, modifying their behavior, decreasing inflammatory responses, and facilitating atherosclerotic lesion regression.	[90]
4.	Nanotherapeutics for Drug Delivery	Nanoparticles can be used to transport cardiovascular medicines, improving drug localization within the afflicted tissue and potentially decreasing the drug dosage and systemic exposure.	[91]
5.	Nano-devices for Cardiovascular Treatment	Nano-patterned vascular stents and nano-devices can be used in the treatment of cardiovascular diseases.	[92]
6.	Nanotherapeutics for Diagnosis	Implantable nano-electronic biosensors and other nanotherapeutics can be used for diagnosis in cardiovascular diseases.	[93]
7.	Nanotherapeutics in Medical Imaging	Nano-platforms can be used as contrast agents in CT imaging to visualize and identify particular cardiovascular diseases, and in ultrasound imaging to target specific vascular-related indicators.	[94]

**Fig. 5:**
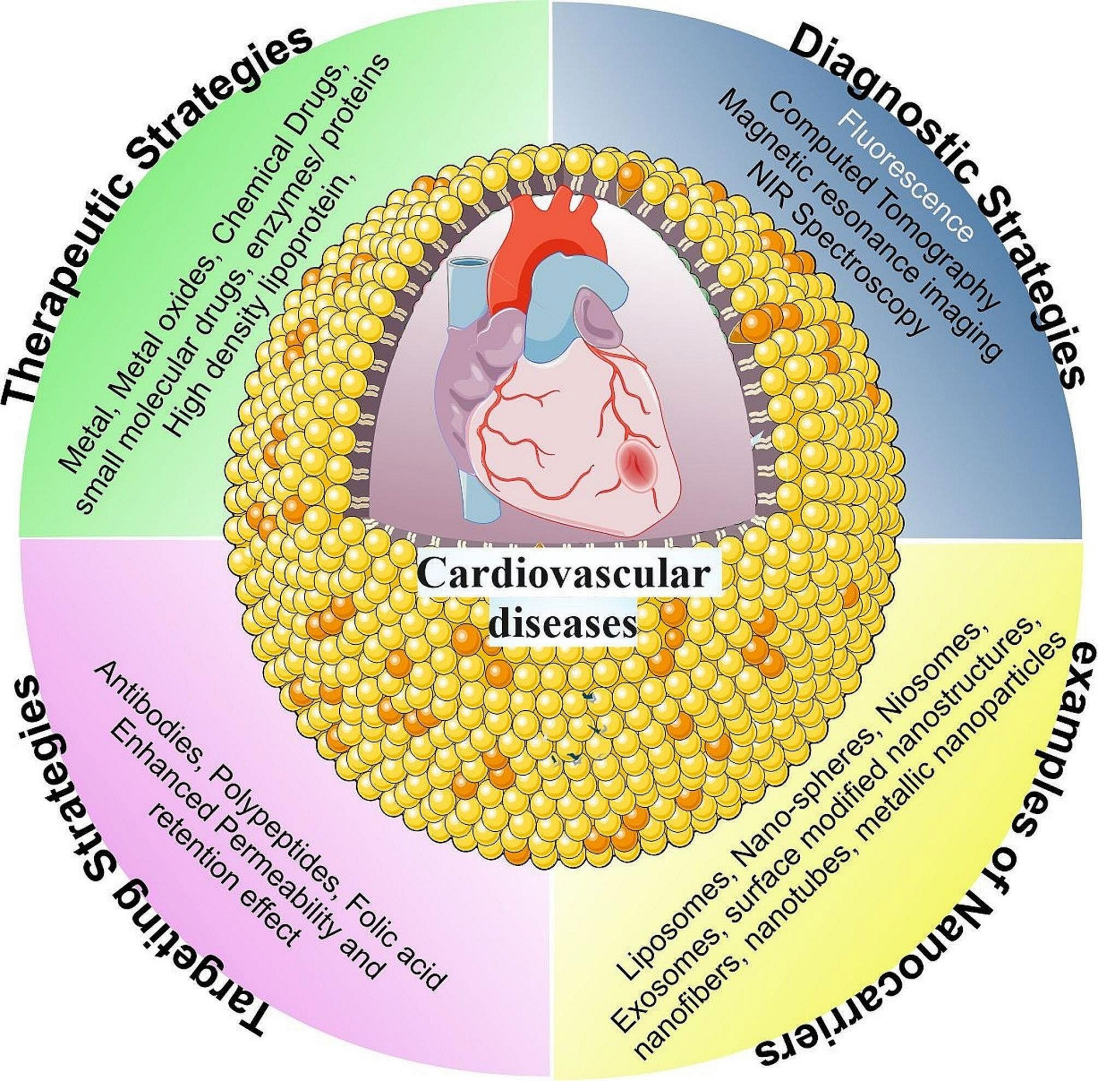
Overview of the diverse applications of nanoparticles as nanotherapeutics, emphasizing their roles in targeted drug delivery, diagnostic imaging, controlled release systems, personalized medicine, and the exciting frontier of emerging applications

A heart transplant is the ultimate treatment for chronic heart diseases, particularly myocardial infarction. However, this may not be possible due to autoimmune disorders, organ rejection risks, and donor shortage. Recently, there has been a greater focus on biomimetic materials based on nanotechnology for the creation of scaffolds [95]. These nanomaterial-based scaffolds aid in tissue regeneration and repair mechanically, electromagnetically, and physically. Moreover, it help tissue function and repair, they can seed cells at the location of an injury or deteriorating tissue [95]. The problems with the currently used conventional stents are being investigated in relation to nano-polymeric-coated biodegradable stents. These new stents have the potential to lower the rate of platelet adhesion while simultaneously improving medication release characteristics. Stents that are anti-thrombogenic and compatible with blood are being made using nanocomposite polymers, such as poly(lactic-co-glycolic acid) (PLGA), polycaprolactone (PCL), and polyhedral oligomeric silsesquioxane poly-(carbonate-urea) (POSS-PCU) [96]. There is a lot of research being done on nano-medicines with various properties and compositions to cure CVDs. These comprise dendrimers, hybrid nano-systems, poly (ethylene glycol)-ated (PEGylated) nano-spheres, liposomes, niosomes, exosomes, surface-modified nanostructures, nanofibers, nanotubes, metallic nanoparticles (NPs), and immune-modified nano-shells [97], mentioned in Fig. 5. Detailed overview of the various applications of nanotherapeutics in cardiovascular diseases is presented in Table 1.

#### Targeted drug delivery system

The process of giving a patient medication in a way that concentrates it more in certain areas of their body than others is known as targeted drug delivery. The goal of targeted drug delivery is to lower the relative concentration of the drug in the other tissues while increasing it in the targeted tissues [98]. This increases the drug’s effectiveness while reducing side effects. This increases the drug’s effectiveness while reducing side effects. Drug targeting is the process of delivering medications to certain parts of the body, such as organs, receptors, or other specified areas [99]. The focused delivery of the medicine with a desired differential distribution would spare the rest of the body, hence reducing overall toxicity and retaining therapeutic advantages [100]. Some of the recent approaches that fall under the category of this system are described below:

One of the approaches is a quantum dot, which is a semiconductor nanostructure that restricts the movement of bound pairs of conduction band electrons and valence band holes, or excitons, in all three spatial directions [101]. Their theoretically high quantum yield, quantum dots are especially important for optical applications. One of the most attractive options for solid-state quantum processing and diagnosis is the capacity to alter the size of quantum dots, which has various benefits [102]. Another one is transdermal drug delivery systems, which use topically applied medications in the form of patches to release medications at a set, controlled rate for systemic effects. A transdermal drug delivery device offers a different method of delivering medication and can have either an active or passive design [103]. One technique used in biotechnology to distribute drugs is called folate targeting. It entails the molecule/drug being attached to the vitamin folate (folic acid) to create a folate conjugate. Folate-drug conjugates also bind strongly to the folate receptor protein (FR), which is frequently expressed on the surface of many human malignancies and causes cellular uptake via endocytosis [100]. DNA fragments and circulating tumor cells in the blood can be found using liquid biopsy imaging, a non-invasive technique. It is essential for detecting cancer early, tracking therapy response, and determining the least amount of disease remaining [104].

#### Nano-platforms in medical imaging for cardiovascular disease detection

Using nano-platforms as contrast agents in CT imaging to visualize and identify particular disease, like cardiovascular disease, by improving the contrast between different tissue types [105, 106]. More specifically, nano-platforms have demonstrated potential in enhancing the visualization of atherosclerotic plaques, deposits of fat, cholesterol, and other materials that can build up in arteries and increase the risk of heart attacks and strokes. Nano-platforms that specifically target atherosclerotic plaques, which can be difficult to detect with traditional CT imaging, can increase the sensitivity and specificity of CT imaging for diagnosing early-stage CVD [107].

One safe and practical medical imaging method that produces images in real-time is ultrasound imaging. The creation of nano-ultrasound imaging materials that can target particular vascular-related indicators has been facilitated recently by breakthroughs in nanotechnology [108]. Perfluorooctyl bromide was used in a study as an ultrasound contrast agent to obtain MR and ultrasound images prior to and during the injection of nano-capsules into atherosclerotic rats [109]. According to the findings, the targeted and control groups did not exhibit echogenicity on ultrasonography, compared to the targeted group. Furthermore, in the targeted group, the nano-capsules considerably decreased the MRI contrast-to-noise ratio; however, this difference was not observed in the non-targeted or control groups [109]. These days, MRIs are a crucial diagnostic tool for CVDs. High-resolution pictures of the heart and the surrounding vasculature are provided by MRI, which enables noninvasive evaluation of the anatomy, function, and tissue characteristics of the heart. One of the special uses of MRI in CVD is the application of parametric mapping techniques, including T1 and T2 mapping, which may quantify changes in tissue composition and reveal small abnormalities that could be missed in traditional imaging [110]. A non-invasive imaging method called positron emission tomography (PET) creates three-dimensional (3D) images of biological processes in the body by using trace amounts of radioactive tracers. PET imaging is utilized in CVD to evaluate the heart’s and blood vessels’ cellular activity, metabolism, and blood flow [111]. Nano platforms are thought to be highly promising instruments for CVD PET imaging. Liposomes, dendrimers, and nanoparticles are examples of Nano platforms that have been utilized in PET imaging for cardiovascular disease [112]. These nano platforms have been functionalized using a range of targeted ligands, which can bind to certain biomarkers or receptors that are overexpressed in CVD. Examples of these ligands include antibodies, peptides, and aptamers. These nano platforms can be used to conjugate chelating agents to provide high-affinity binding for a range of radioactive or non-radioactive heavy metal ions used in imaging modalities, including ^111^In, ^99^mTc, ^68^Gd, ^67^Ga, and others. They can also be used to encapsulate hydrophobic imaging components [113].

#### Types of HDL mimetic therapeutics

A family of naturally occurring nanoparticles called HDL, is made up of a dynamic mixture of proteins, lipids, and other biomolecules [114]. The earliest discovery of the correlation between HDL and disease incidence occurred in the 1950s when an elevated risk of cardiovascular disease was associated with a lipoprotein cholesterol content. This was further supported by the comprehensive Framingham research conducted in the 1960s [115–117]. The primary anti-atherogenic characteristic of HDL is thought to be its capacity to enhance cellular cholesterol efflux and reverse cholesterol transport (RCT). This has led researchers to wonder if direct infusion of reconstituted Apo lipoprotein A-I (ApoA-I), the main protein component of HDL, can lessen coronary events in people with CVD [118]. The most promising alternative option for modifying the structure of HDL is to use peptidomimetics instead of full-length apo-AI [119]. Early human trials revealed that 4 F offers additional benefits in people receiving lipid-lowering medications such as statins. Other research has reported on apoA-I mimetics as promising therapies for the treatment of obesity and the decrease of adipose tissues [120–122]. ApoA-I mimetics have also been linked to improvements in insulin resistance, an increase in adiponectin levels, and a reduction in oxidative stress in chronic kidney disease. A primary reason for the anti-atherogenic properties of apo-AI mimetic is their strong affinity for the ABCA1 transporter, which facilitates reverse cholesterol transfer via triggering JAK-2 in macrophages [123, 124] although their anti-inflammatory effect is most likely accomplished by binding to oxidized lipids and inhibiting the generation of pro-inflammatory cytokines [125–127]. To imitate the natural activity of HDL, recent attempts in building artificial rHDLs have broadened to use peptide mimetic rather than full-length apo-AI. To that objective, several lipid-peptide complexes with varying lipid compositions and lipid-to-peptide ratios were produced [128]. Nano disc HDL particles are frequently formed by apo-AI mimetic peptides binding to lipids, which, like real HDL, have been demonstrated to reduce atherosclerosis by removing cellular cholesterol and phospholipids through ABCA1 transporter-dependent pathways [129, 130]. For the purpose of visualizing atherosclerotic plaques, apo-AI mimetic peptide-based rHDL particles have also been thoroughly investigated. For simultaneous MRI and fluorescence imaging, Gd-chelate complexes and rhodamine were used to functionalize a synthetic HDL nanodisc expressing the apo-AI mimetic peptide 37pA. Following the in vivo administration of functionalized HDL Nano discs, an analysis between apo E-/- and wild-type mice showed a notable increase in the MRI signal in the apo E-/- mice’s aortic plaques but no discernible signal in the aortas of the wild type mice [131]. Reconstituted rHDL is a form of nanoparticle that mimics the structure and function of HDL. The structural components of native HDL, including phospholipids, Apo lipoproteins, and cholesterol, are reconstituted to create rHDL nanoparticles. This method enables exact control of nanoparticle composition and characteristics, allowing rHDL to be tailored for specific therapeutic applications. rHDL nanoparticles have been demonstrated to be useful in the treatment of a wide range of ailments, including cardiovascular disease, cancer, and neurological disorders. rHDL nanoparticles, for example, have been utilized to carry medications to tumors, where they can kill cancer cells while not hurting healthy cells. In neurodegenerative disease such as Alzheimer’s, rHDL nanoparticles have also been employed to shield nerve cells [132].

The applications of nanotherapeutics in cardiovascular disease encompass targeted drug delivery systems, imaging and diagnostic innovations, and various types of HDL mimetic therapeutics. Targeted drug delivery focuses on concentrating medication in specific body areas to enhance effectiveness and reduce side effects. Approaches include quantum dots for optical applications, transdermal drug delivery systems, and folate targeting. Imaging and diagnostic innovations utilize nanoplatforms as contrast agents in CT imaging, enhancing visualization of atherosclerotic plaques. Ultrasound imaging employs nano-ultrasound materials targeting vascular indicators. Nanoplatforms in MRI and PET imaging offer non-invasive evaluation of cardiovascular function and cellular activity. HDL mimetic therapeutics explore reconstituted Apo lipoprotein A-I (ApoA-I), apo-AI mimetics, and rHDL nanoparticles, demonstrating the potential for treating cardiovascular and other diseases. The comprehensive review aims to provide insights into these applications, bridging gaps in current literature.

### Mechanism of action

#### HDL nanotherapeutics with monocytes and macrophages

HDL mimetic nanotherapeutics are intended to target monocytes and macrophages, which are important players in atherosclerosis and inflammation. These nanotherapeutics work by lighting intracellular signaling networks as well as complex recognition and binding mechanisms. HDL mimetic nanoparticles are engineered to identify and bind to certain receptors on monocytes and macrophages, including CD40 and tumor necrosis factor receptor-associated factor 6 (TRAF6). This contact enables nanoparticles to be ingested by cells and exhibit their therapeutic effects [141]. Once within the cells, HDL mimetic nanoparticles can influence a variety of intracellular signaling pathways, including the CD40-TRAF6 pathway. In atherosclerotic plaques, this modulation can result in the inhibition of monocyte recruitment, the reduction of lesional macrophages, and the suppression of macrophage proliferation and death. HDL-mimetic nanoparticles can help with cholesterol efflux from foamy macrophages to the liver, which can help reduce atherosclerotic plaques. This mechanism is required for HDL’s antiatherogenic activity [141]. Some HDL mimic nanoparticles have been engineered to be very selective for M2 macrophages, allowing them to accumulate specifically in atherosclerotic plaques. This selectivity can assist in reducing the negative effects of traditional medicines, such as liver X receptor agonists, which can induce severe hypertriglyceridemia and liver steatosis [142].

### HDL mimetic nanotherapeutics in cardiovascular health

HDL mimetic nanotherapeutics have emerged as a viable strategy in the treatment and prevention of cardiovascular disorders, particularly in the treatment and prevention of foam cell formation and the orchestration of anti-inflammatory responses. Macrophages and monocytes are important mediators of vascular inflammation, such as in coronary artery disease. On mononuclear phagocytes, HDL nanoparticles have strong anti-inflammatory properties that provide new opportunities for the development of nanotherapeutics to maintain vascular integrity [143]. These nanotherapeutics are at the core of many disease pathophysiologies because of their ability to interact with a wide variety of immunological and structural cells. Acute coronary syndrome patients in phase III clinical trials using HDL-based nanoparticles are exhibiting encouraging outcomes, and the formulation and components of these particles have undergone substantial development [133]. HDL mimic nanotherapeutics attempt to reduce the lipid burden in atherosclerotic plaques by increasing cholesterol efflux from foam cells to the liver. Furthermore, these nanotherapeutics orchestrate anti-inflammatory responses by modulating monocyte recruitment to the vessel wall and decreasing inflammatory cytokine expression, restoring vascular integrity and mitigating the inflammatory processes associated with cardiovascular disease. These nanotherapeutics can restore functional HDL particle counts and increase HDL functionality, providing patients with cardiovascular disease with supplementary therapeutic methods beyond decreasing atherogenic lipoproteins [133, 143].

### The role of HDL nanoparticles in atherosclerosis

HDL nanoparticles have become a viable option for addressing atherosclerosis because of their distinct features. HDL has a vital function in the process of reverse cholesterol transport, which involves moving excess cholesterol from tissues in the body, such as atherosclerotic plaques, to the liver for elimination [144]. Research has shown that HDL nanoparticles may efficiently engage with atherosclerotic lesions, enhancing the elimination of cholesterol and encouraging the regression of plaque. The process is facilitated by different receptors found on macrophages in the plaque, including scavenger receptor class B type I (SR-BI), ATP-binding cassette transporter A1 (ABCA1), and ATP-binding cassette transporter G1 (ABCG1). These receptors help in removing cholesterol from the plaque and transferring it to HDL particles. In addition, HDL nanoparticles possess anti-inflammatory and antioxidant characteristics, which enhance their therapeutic efficacy in treating atherosclerosis [145]. The utilization of HDL nanoparticles shows potential for attenuating the advancement of atherosclerosis and diminishing the related cardiovascular peril.

Researchers have designed HDL mimetic nanoparticles that imitate the advantageous properties of natural HDL in specifically targeting atherosclerotic plaques. These nanoparticles are designed to replicate the structure and function of natural HDL, enabling them to engage with the same receptors involved in the transport of cholesterol and the regulation of inflammation [146]. An effective method includes using poly (lactic-co-glycolic acid) (PLGA) as a central substance, capable of enclosing hydrophobic medications, while also being changed on the surface to imitate the protein makeup of HDL. Research has shown that HDL mimic nanoparticles have a higher ability to specifically target atherosclerotic plaques, resulting in better delivery of therapeutic medicines. Moreover, the capacity to adjust the physicochemical characteristics of these nanoparticles permits tailoring to precise therapeutic needs [146].

**Fig. 6:**
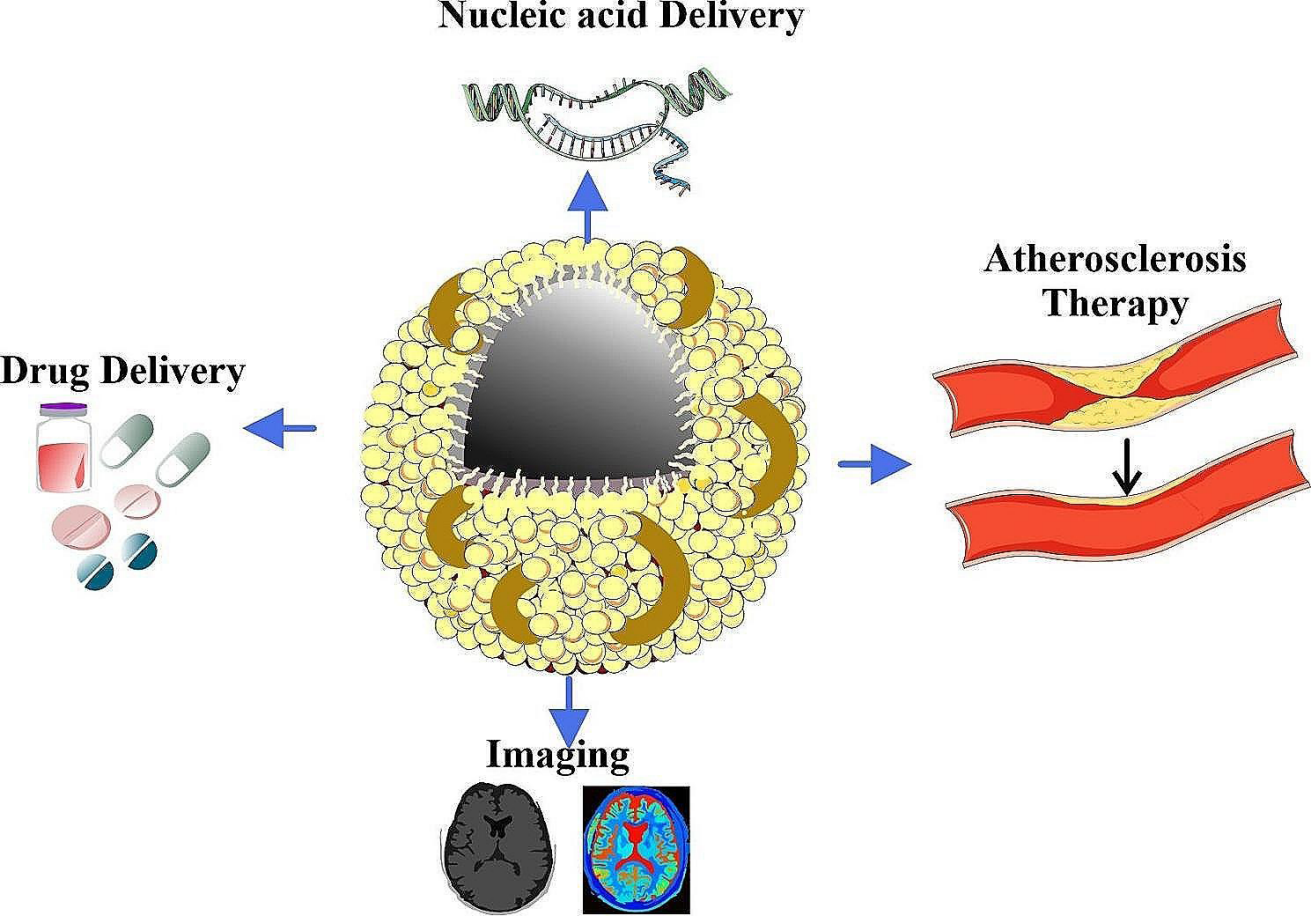
High density lipoprotein nanoparticles as potential therapies and for molecular delivery

HDL nanoparticles have a unique ability to assemble themselves, which enhances their biological activity and effectiveness in treating atherosclerosis. The production of these nanoparticles occurs via the interaction of apolipoproteins, particularly apolipoprotein A-I (apoA-I), and lipid molecules, leading to the creation of discoidal or spherical structures. The flexible structure of HDL assembly allows for the incorporation of different lipid and protein components, offering variety in delivering cargo and recognizing receptors [139]. Furthermore, the adaptable structure of HDL nanoparticles enables them to engage with certain cells, such as macrophages found in atherosclerotic plaques. This contact promotes the removal of cholesterol and regulates inflammatory reactions. Moreover, the capacity of HDL particles to restructure and get extra lipid and protein components significantly amplifies their functional variety and potential for therapy (Figure 6) [139].

### Biocompatibility and efficacy of HDL nanoparticles

Synthetic high-density lipoprotein (HDL) nanoparticles have shown exceptional compatibility with living organisms and effectiveness, making them very appealing for potential use in treating atherosclerosis. These nanoparticles have low levels of toxicity to cells and little potential to provoke an immune response, which makes them compatible with biological systems and reduces the likelihood of unpleasant responses [147]. Moreover, the biodegradable properties of synthetic HDL nanoparticles enable them to degrade and be eliminated from the body in a regulated manner, reducing the risk of long-term buildup and possible harm. Research has shown that artificial HDL nanoparticles efficiently facilitate the removal of cholesterol from macrophages, hinder inflammatory processes, and regulate the stability of plaque. As a result, they provide positive results in animal models of atherosclerosis. Furthermore, the capacity to customize the structure and surface characteristics of HDL nanoparticles allows for the modification of their performance to achieve particular therapeutic objectives, hence improving their effectiveness and accuracy in targeting [148].

### Pre-clinical and clinical studies

#### An overview of clinical trials

Humans were tested for ApoA-I extracted from pooled human plasma since overexpressing ApoA-I produced positive results in animal models of atherosclerosis [149–151]. Since overexpressing ApoA-I had positive outcomes in animal models of atherosclerosis, humans were tested with injections of ApoA-I obtained from pooled human plasma. In theory, this therapy replicates the production and release of immature ApoA-I, which would be the first phase of HDL biogenesis. When injected into the blood, results in pre-beta HDL through lipid incorporation via ABCA1, which subsequently develops into fully formed HDL nanoparticles, very similar to native HDL biogenesis. Research confirmed that it is safe for humans to use. However functional protective readouts in terms of increased cholesterol efflux capacity were equivocal due to the free protein’s rapid catabolism [152]. Following the identification of a mutant form of ApoA-I in Milan, which was subsequently dubbed ApoA-I Milano (ApoA-IM), another method for maximizing the cardio protective advantages of ApoA-I was developed. The variant, which is an illustration of a single nucleotide polymorphism, was identified by the substitution of cysteine for arginine at position 173, which seems to be linked to improved vascular health in carriers [153, 154]. When ApoA-IM was administered to atherosclerotic mice, the amount of plaque decreased significantly, and the resolution of CAD was aided [155, 156]. These encouraging results led to the design of the first randomized controlled clinical study (ECT-216), which provided full-length recombinant ApoA-IM complexed with POPC [157]. The outcomes were positive in terms of plaque volume reduction and CAD regression. A second study found a significant relationship between compensatory vascular elastic remodeling and reduced atheroma volume in a specific and focused manner [158].

### Preclinical studies

Preclinical research into HDL nanotherapeutics has been critical in determining the potential of these novel platforms for treating cardiovascular disorders. The unique properties of natural HDL, such as its role in reverse cholesterol transport and anti-inflammatory effects, have fueled the development of biomimetic nanotherapeutics that seek to imitate and increase these capabilities. Duivenvoorden et al. revealed preferential accumulation of HDL-mimetic Nano carriers in atherosclerotic lesions in a mouse model in the study of bio distribution and pharmacokinetics, stressing their potential for targeted medication delivery to areas of vascular inflammation. This fundamental understanding of the pharmacological action of HDL mimetic nanoparticles aids in the refinement of dosage methods and the establishment of the therapeutic window during the preclinical phase [159].

Furthermore, preclinical models have been useful in determining the therapeutic efficacy of HDL mimic nanotherapeutics in the treatment of atherosclerosis. Pérez-Medina et al. used a mouse model to show that HDL mimic nanoparticles loaded with statins significantly reduced plaque burden and inflammation, highlighting the therapeutic potential of these Nano carriers for atherosclerosis control. These preclinical studies give critical mechanistic insights that will guide the translation of HDL mimic nanotherapeutics into clinical applications [160]. Various examples of studies conducted in this are summarized in Table 2.

**Table 2:** Recent advances made to explore the role of High Density Lipoprotein in cardiovascular diseases

S.No	Recent studies conducted in this area	References
1	Artificial high-density lipoprotein-mimicking nanotherapeutics for the treatment of cardiovascular diseases	[133]
2	Plaque-hyaluronidase-responsive high-density-lipoprotein-mimetic nanoparticles for multistage intimal-macrophage-targeted drug delivery and enhanced anti-atherosclerotic therapy	[134]
3	A Current Update on the Role of HDL-Based Nanomedicine in Targeting Macrophages in Cardiovascular Disease	[72]
4	Roles of reconstituted high-density lipoprotein nanoparticles in cardiovascular disease: a new paradigm for drug discovery	[135]
5	HDL-Mimetic PLGA Nanoparticle to Target Atherosclerosis Plaque Macrophages	[136]
6	Nanotechnology for cardiovascular diseases	[137]
7	Nanoimmunotherapy to treat ischaemic heart disease	[138]
8	Artificial High Density Lipoprotein Nanoparticles in Cardiovascular Research	[139]
9	Application of targeted therapy strategies with nanomedicine delivery for atherosclerosis	[140]
10	High-density lipoprotein mimetic nanotherapeutics for cardiovascular and neurodegenerative diseases	[132]

The constraints and potential related to the clinical implementation of HDL mimic nanotherapeutics are substantial. Nanotherapeutics that mimic HDL have shown potential in preclinical investigations as a treatment for cardiovascular ailments. These innovative platforms seek to replicate and amplify the distinctive characteristics of natural HDL, including its function in reversing cholesterol transport and its anti-inflammatory effects [161]. Nevertheless, there are other obstacles that must be overcome prior to the introduction of these innovative formulations onto the market. The problems include the creation and use of particles at the nanoscale, improvement of dosing techniques, determination of the optimal therapeutic range, and resolution of regulatory and safety concerns.

The use of HDL mimic nanotherapeutics in clinical settings offers possibilities for precise drug delivery to regions of arterial inflammation. The nanotherapeutics possess the capability to control the recruitment of monocytes and the polarization of macrophages, therefore hindering the advancement of atherosclerosis. Moreover, the advancement of nanotherapeutics equipped with precise targeting abilities enables them to specifically engage with monocytes and macrophages, effectively using the innate anti-inflammatory properties of HDL [90]. The focused alteration of monocyte and macrophage activity aligns with HDL’s ability to defend against atherosclerosis and might contribute to the stabilization of atherosclerotic plaques. The use of HDL mimetic nanotherapeutics shows great potential for treating cardiovascular diseases. However, it is crucial to overcome the challenges related to the development and use of nanosized particles. Additionally, a deeper understanding of the specific molecular pathways and long-term effects of these nanotherapeutics on monocytes and macrophages is necessary to optimize their effectiveness as treatments [162].

### Challenges and future perspectives

#### Nanotherapy horizon: addressing development and implementation challenges

Overcoming various challenges, particularly with nanosized particles, is crucial before novel formulations can realistically enter the market in ten to twelve years. Complying with good manufacturing practice (cGMP) regulations for large-scale production is one of the several obstacles in the translation of laboratory findings to the clinic [163]. As currently the case with CSL111 and CSL112, stringent quality control requirements must be met to guarantee that apo-AI is purified from human plasma to create HDL mimetics and that the plasma product is devoid of immunogenic or infectious impurities. Multi-step expression and purification techniques are necessary when using recombinant proteins, and these procedures are costly and time-consuming. Considering limitations, the benefits of entirely synthetic goods over organically derived components include lower production costs and greater freedom in adjusting the structure of HDL. In order to achieve translation scalability, production measures such as yields and sample quality must also be considered [164, 165]. Another issue that needs to be addressed for the upscaling process is that the rHDL particles, which are formed when peptides, lipids, and medicines self-assemble, necessitate extensive and time-consuming purification and homogenization operations. The perfect control of physicochemical particle properties is a fundamental problem in the synthesis and large-scale manufacture of self-assembled nanostructures such as rHDL. The results of the assembling process may be affected by the production method, composition, and concentration, presence of excipients, salts, pH, or temperature. To ensure batch-to-batch repeatability across laboratories, standard operating procedures (SOPs) and defined quality requirements for chemical, physical, and structural particle parameters must be developed. For cGMP production in clean pilot plants, quality control (QC) and quality assurance (QA) standards are substantially stricter, necessitating in situ quality control measures of the manufacturing process and, as a result, product stability [166–168]. From the outset of the development of nanomedicine (NNM), it is imperative to take into account the relationship between disease pathophysiology and disease heterogeneity in humans, as well as the significance of the physicochemical properties of various NNMs in surmounting biological barriers to facilitate enhanced targeting to diseased tissue and decreased accumulation in non-target organs. Comparatively, less research has been done on the relationships between patient disease heterogeneity and NNM behavior in specific clinical applications. These are most likely the primary reasons why potential NNMs are unable to move forward with clinical studies [169].

### Future perspectives

Future developments in HDL mimetic medicines have great potential to improve methods for treating and preventing cardiovascular disease. The goal of ongoing research is to improve and optimize HDL mimetic nanoparticle design in order to increase their therapeutic efficacy and expand their range of applications. Investigating cutting-edge nanomaterials and engineering methods to enhance drug loading capability, stability, and tailored drug release from HDL mimic platforms is one important avenue.

Given the abundance of evidence supporting the positive effects of HDL on monocytes and macrophages [35]. Numerous clinical trials sought to translate HDL infusion therapy successfully. Much research has focused on the role of HDL in cardiovascular disease. Current findings on loaded and modified HDL nanodiscs demonstrate the need for ongoing optimization efforts to enhance the pharmacological characteristics of synthetic HDL nanoparticles, including half-life and targeting precision. Polyethylene glycol (PEG) insertion into synthetic HDL mimetics, for example, boosted cholesterol mobilization in rats [170]. Polylactic-co-glycolic acid (PLGA) supplementation to sHDL is another modification that has been studied. In addition to atheroprotective properties, PLGA supplementation of sHDL has been found to confer selective localization in atherosclerotic plaques [136]. ApoA-I nanoparticles with metal cores that imitate HDL activities are also a viable diagnostic tool for detecting atherosclerotic plaques. It has been demonstrated that gold-based sHDL improves reverse cholesterol transport channels and plaque analysis. ApoA-I-based gold sHDL stabilized cholesterol esters in the core, enabling long-term LCAT activation and continuous cholesterol efflux. The incorporation of gold and iron into sHDL provided diagnostic benefits in computed tomography (CT) scans by increasing the signal-to-noise ratio during detection. In addition to atherosclerosis, HDL mimics show encouraging results in mouse models of sepsis and acute respiratory distress syndrome [171, 172].

## Conclusion

HDL mimetic nano-therapeutics offer a potential approach to treat cardiovascular diseases by targeting monocytes and macrophages. However, the development of these nano-therapeutics faces challenges such as the identification of suitable ligands, optimization of drug loading and release profiles, and ensuring specific targeting to monocytes and macrophages. Additionally, the translation of these nano-therapeutics into clinical applications requires further research and development. To overcome these challenges, researchers should focus on investigating the mechanisms of action of these nano-therapeutics and their effects on monocytes and macrophages in various cardiovascular conditions. They should also identify suitable ligands and develop strategies for specific targeting to monocytes and macrophages. Furthermore, optimizing drug loading and release profiles to ensure efficient and controlled drug delivery is crucial. Conducting preclinical and clinical trials to evaluate the safety and efficacy of HDL mimetic nano-therapeutics in treating cardiovascular diseases is also necessary. In the future, more research is needed to optimize the design of HDL mimetic nano-therapeutics and understand their mechanisms of action in detail. With continued research and development, HDL mimetic nano-therapeutics show promise approach in targeting monocytes and macrophages for improved cardiovascular care and ultimately improving patient outcomes.

## Data Availability

No datasets were generated or analysed during the current study.
